# A biocompatible supramolecular hydrogel mesh for sample stabilization in light microscopy and nanoscopy

**DOI:** 10.1038/s41598-024-76661-x

**Published:** 2024-11-25

**Authors:** Marko Lampe, Bart Dietrich, Joanna Wnetrzak, Tom Waring, Gareth Lycett, Marisa M. Merino, Dave J. Adams, Marco Marcello

**Affiliations:** 1 Advanced Light Microscopy Facility, EMBL, Meyerhofstr. 1, 69117 Heidelberg, Germany; 2 School of Chemistry, University of Glasgow, Glasgow, G12 8QQ UK; 3 Centre for Cell Imaging, Institute of Systems, Molecular and Integrative Biology, Liverpool, L69 7ZB UK; 4 Liverpool School of Tropical Medicine, Liverpool, L3 5QA UK; 5 Molecular & Clinical Cancer Medicine, Institute of Systems, Molecular and Integrative Biology, Liverpool, L69 7ZB UK

**Keywords:** Imaging, Microscopy, Biological techniques, Biotechnology, Chemistry, Materials science, Optics and photonics

## Abstract

Most embedding media for live and fixed samples were not designed for microscopy and have issues including long polymerization times, peak of toxicity toward the sample during the sol-gel transition, and irreversibility of this transition. Gels derived from biological sources are widely used in microscopy, but their precise composition is ill-defined and can vary between batches. Non-physiological temperatures and/or specific enzymatic solutions are often needed to revert the gel back to the sol state to allow sample recovery. Recovering the sample undamaged is important for multiple purposes, from the ability to release a living organism back into its environment and re-observe it at a later stage, to interrogating the sample once freed from the gel after imaging. We describe a supramolecular hydrogel that enables the observation of small living organisms using light microscopy, with simple sample recovery through vigorous pipetting with water. The organisms can be recovered alive and capable of further development into adulthood, which represents a significant advancement, as most other matrices require release conditions such as heating, the addition of chemicals, or mechanical disruption, which can damage or kill the embedded organisms. Furthermore, the gel is compatible with super-resolution multi-colour STED nanoscopy.

**Supplementary Information:**

The online version contains supplementary material available at 10.1038/s41598-024-76661-x.

## Introduction

In recent years, there have been significant efforts from academia and companies alike to create new kinds of biosynthetic hydrogel scaffolds that can be used in various applications including cell culture, tissue engineering and microscopic sample preparation. While a range of innovative tools was developed in the cell culture^1,2^ and tissue engineering fields^3,4^, in the microscopy field the most commonly used gels used for mounting samples are still products that were originally developed for other areas. A good example is agarose, where most formulations commercially available were designed for electrophoresis or cell culture^5^. Hence, they were not optimized for microscopy imaging. In parallel, in the last decade new microscopy techniques such as Light Sheet Fluorescence Microscopy and Optical Projection Tomography^6,7^ have changed the landscape of the bio-imaging world. Thanks to these innovative tools, a new kind of volumetric imaging was introduced where the sample, no longer constrained in the traditional coverslip/slide geometry, could be mounted into a three-dimensional matrix. Once embedded in this matrix, the sample could be translated and rotated in all spatial directions, different views could be acquired and merged, and several structures ranging from microns to centimetres could be imaged with a precision of optical sectioning that was previously unattainable. The field is now sufficiently mature to develop new kinds of hydrogels with improved physicochemical characteristics optimized for the use in 3D microscopy, but with features that can also make them useful in other sectors.

There are a number of hydrogels used to immobilize samples in microscopy, from gellan gum^8^, which is sold under a number of brand names such as Phytagel™ and Gelrite™, to methylcellulose^9^ and CyGel^10^. The ideal scaffolding for sample preparation in light microscopy should exhibit definite physicochemical properties, the most important probably being optical clarity to minimize light dispersion for the photons entering the sample (illumination) and those exiting (signal). Low toxicity is a crucial property of the ideal gel for microscopy as the mechanisms triggering gelation can have a very different impact on different samples. There are a number of potential issues with chemical crosslinking. The toxicity of the gel can be due to chemical groups in the polymer that interact unfavourably with the metabolism of the living, embedded sample. For example, glutaraldehyde can be used to crosslink amines on a polymer, but also reacts with lysine residues on embedded materials^11^. Similarly, a crosslinking agent that reacts with carboxylic acids can react with aspartic acid or glutamic acid residues^11^. Photocrosslinking can be used, but often requires the presence of toxic initiators and the monomers used are often toxic; light can also damage organisms depending on wavelength^12^. However, even in the absence of toxic chemicals in the gel, one could expect a peak of toxicity in the hydrogel timed at the moment of the sol-gel transition. To trigger the gelation and cause all the crosslinking between the polymers to give to the gel the desired stiffness, one or more physical parameters must change abruptly. Quite often this parameter is the temperature, like in the case of agarose, but it could also be the pH or the concentration of ions in solution (normally Ca^2+^ or Mg^2+^). Unfortunately, this sharp change in these physical parameters normally causes a stress or even damage to the living specimen, which in the worst cases is irreversible and leads to sample death. Even after the dramatic sol-gel transition, the gel must still have satisfactory biocompatibility parameters, such as ability to exchange gases, low cytotoxicity and low stiffness of the mesh to allow for normal development.

Agarose has been in use in biology and microscopy laboratories for decades and its physical properties are precisely characterized^13^. However, if retrieval of the sample from the matrix is crucial, agarose is not an optimal choice, as the high temperatures needed to disrupt the matrix will in most cases damage the sample. Therefore, there is an increasing need for hydrogels with a reversible transition between the gel- and the sol-phase^14^ for example in case of further processing the sample with -omics approaches. An ideal hydrogel used in fluorescence microscopy should obviously have low autofluorescence. Some recent commercial formulations tend to show this issue^15^. The kinetics of the crosslinking tag of the polymers that confers stability to the gel also has a particular importance for the ease of handling of the gel with different samples. Such a transition should be completed with timing compatible with the standard pipeline of imaging procedures. The transition from sol to gel for commonly used embedding media depends on several factors, including ambient temperature and media concentration, and it ranges from few minutes (alginate, Phytagel) to several hours (methylcellulose) or even one day (ProLong Gold) to fully cure. Our hydrogel can be placed among the rapid solidifying mounting media, which is preferrable in case of time-sensitive experiments, and to minimize sample displacement. A hydrogel for microscopy observation of living samples should also have an inherently low toxicity. The gel stiffness needed to embed the sample should not impair the development of the sample and allow some degree of sample movement^16^. Finally, the mesh of the gel should not be so tight as to hinder gas exchange and prevent the development of the embryo^17^. We thereby clarify that our hydrogel is meant to be a sample stabilizer and not a growth matrix for cell culture.

Here, we have designed a supramolecular hydrogel consisting of a reversible fibrillar network specifically for microscopy, capable of stabilizing the sample during imaging but with improved biocompatibility to grant the correct development of the embedded organism. The organisms of choice for testing the biocompatibility of our gel were *Aedes aegypti* and *Drosophila melanogaster*. The mosquito species is a vector of human disease-causing viruses, which include dengue fever, yellow fever and Zika. *Drosophila* is the most used model for developmental biology. Here, we have developed the first mechanically stable and biocompatible hydrogel mesh in which samples can be imaged and from which the insects can be extracted and reach the adult stage with a very high survival rate and without developmental defects. Furthermore, this hydrogel showed excellent performance with Stimulated Emission Depletion (STED) nanoscopy of fixed samples, where homogeneity and mechanical stability as well as optical and chemical inertness are important features, confirming its full compatibility for demanding microscopy applications. In addition, the hydrogel is inexpensive, easy to store and to prepare and fast in the sol-gel transition.

## Results

### Gel formation

**Fig. 1:**
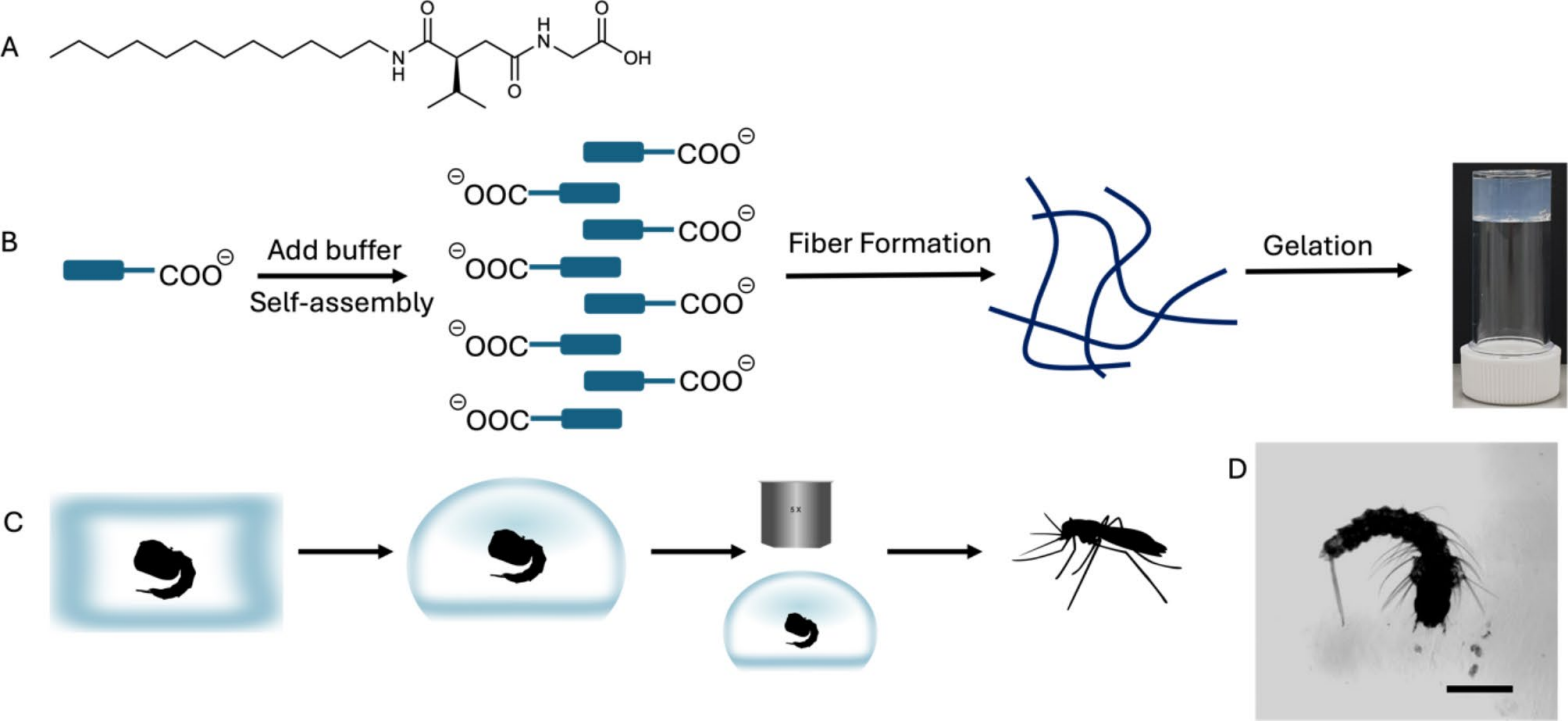
Design, embedding and release of the sample from the hydrogel. (**A**) Chemical structure of the gelator used here. (**B**) Cartoon showing the gel formation. The initially soluble molecule is salted out by addition of buffer to lower solubility, inducing self-assembly to form fibers, which entangle to form a network. The photograph of a gel formed from this gelator showing the transparency. (**C**) Workflow for microscopy: after addition of the buffer the pupa is dipped into the hydrogel in its sol state on the microscope support chosen for imaging. Thanks to the quick sol-gel transition the pupa is immobilized in the gel. The pupa can undergo microscopy observation. After release from the hydrogel, the live pupa can be returned to the insectarium to reach adulthood. (**D**) Mosquito larva embedded in the hydrogel imaged in transmitted light. Larva of mosquito embedded in hydrogel imaged on a LSM 510 (Zeiss, Jena, Germany) with a 2.5 × NA 0.12 objective. Scale bar:1 mm.

 The hydrogel we developed is based on a variant of a gel from a previous publication^18^, which describes self-assembling biomolecular fibrillar networks. Two of us have recently found that this material can be used for encapsulation of proteins^19^ and as part of this work observed the high transparency of this material. Gels are formed by the self-assembly of small molecules (the hydrogelator) into fibers which entangle. Analogously to the case of agarose developed for electrophoresis we have an “off-label” application of a tool that was not specifically devised for microscopy. The hydrogelator is prepared from amino acids by *N*-acylation with succinic anhydride and amide formation (the chemical structure of the gelator is shown in Fig. 1 A). A precursor solution was prepared at pH 8 (see “Methods” section). The gelation was triggered by lowering the pH of the precursor solution by adding a buffer. This results in a decrease in solubility of the gelator and self-assembly into a network (Fig. 1 B). Initially, we used PBS. The gelation time under these conditions was relatively slow, occurring within one hour at room temperature. The final pH of the hydrogel was 8.5, outside of the suitable range with respect to physiological values. Different buffers were examined to decrease the gelation time and render the gel more amenable to use to embed samples for microscopic imaging. The buffer giving the best results was Tris HCl 1.5 M pH 6.8, resulting in a gelation time of within 5 min and a final pH of 7.3 (See Supplementary Fig. S1 online).

We used rheology to precisely characterize the viscoelastic properties of the hydrogel (See Supplementary Fig. S2 online). Time sweeps confirm the fast gelation process, with the storage modulus (G′, associated with the solid-like properties) dominating over the loss modulus (G″, associated with the liquid like properties) in around 5 min; that G′ is greater than G″ shows the solid-like nature of the sample) in around 5 min. There is a further evolution with time with both G′ and G″ slowly decreasing, although G′ always dominates over G″. In all cases, it is worth noting that the material is not a true gel in terms of the ratio between G′ and G″. However, for the application required here, the material exhibits suitable properties, and, for the ease of discussion, we refer to it as a gel throughout.

An important feature for hydrogels embedding microscopy samples is that the light should be able to travel through the specimen without much absorbance or scattering. We completed the gel characterization by examining its most important photophysical property, transmittance, comparing it against agarose. We tested this property qualitatively and quantitatively with a spectrofluorimeter, consistently finding higher transmittance in our hydrogel as compared to agarose (See Methods and Supplementary Fig. S3 and S4 online). Our hydrogel presents a stable refractive index which, matched with that of the sample, is also crucial for an improved quality of the image. Our gel formulation displays a refractive index of 1.37 (532 nm) which is in range of the majority of biological specimens (1.33–1.51)^20^. Additionally, even gels transparent to the human eye can be autofluorescent under imaging conditions in a fluorescent microscope due to the presence of specific chemical groups in the polymers. Our hydrogel shows negligible fluorescence background at all the wavelengths used (from 350 nm to 631 nm, Figure S4 and S5).

### Biotoxicity

 Mounting conditions for non-invasive imaging of delicate, living samples are crucial, and the existing, commonly used gels for immobilizing the samples have several limitations. The main limitation is the impossibility of recovering the sample after the imaging session. While it is certainly true that there is no universal mounting technique that applies to any organism/organ of interest through any phase of development and for any duration of the whole experiment, we tested our hydrogel to image *Aedes aegypti* larvae (see Fig. 1C) and to follow the development of mosquito pupae following gel entrapment. As discussed elsewhere^21^, there are several ways to assess the toxicity of an experiment for a live sample, depending on the sample and on the technique used. Briefly, we used the most stringent criterion for assessing toxicity for our gel, which involves not only evaluating the mere survival of the sample right after the experiment but also observing the subsequent growth of the sample to normal adulthood.

Immediately after triggering gelation by addition of the buffer, each pupa (29 overall) was individually dipped into the liquid. When transition was completed, each pupa remained embedded in the hydrogel for one hour at 27 °C. After this time, the pupae were freed from the mesh by vigorously pipetting some distilled water up and down (Fig. 1C-D, see Methods and Video S2).

This is possible because of the low strain at which the gels break (see Fig. S2, Supporting Information), which differs from other gels such as agarose. The breaking of the gel is due to the mechanical action breaking the network which is not permanently crosslinked, but rather held together by entanglement. All the pupae were then returned to the vivarium. The vast majority was raised to adulthood and no difference in the morphology and survival rate of the adult insects was observed with respect to the controls grown in the vivarium (86.2% survival rate, please see Figs. S11 and  S12).

It is worth noting that a negative control of pupae that never left the vivarium and never came in contact with the gel, with optimal conditions of light, humidity and temperature and without any external manipulation throughout their development, had a survival rate of 93.3% (Fig. S11).

We then added a further control, with conditions of increasing biotoxicity for the live samples: pupae this time were embedded in a 6-well plate and transported in a different building on campus to be imaged. After imaging pupae were returned to the original building in the vivarium, freed from the gel and raised to adulthood. In this case mortality was much higher, with only 13.3% of survival rate (Fig. S11).

For this last group, pupae were embedded in the gel for two hours in total, because the setup of the high-throughput microscope (Cell Discovery 7, Zeiss, Jena, Germany) needs about one hour preparation to find focus and spatial coordinates of every sample before effective start of the one-hour timelapse, with an image every five minutes (Fig. S9).

When comparing the survival rates of insects under different conditions, one must consider how we define combined toxicity. Together with gel embedding, temperature and humidity shock as pupae are transported between buildings, the biggest factor is probably the manipulation of the pupae to transfer them from insectarium to the different containers where they are imaged and back.

The pupal stage involves significant physiological changes. The pupa’s outer layer (cuticle) is rigid in the case of drosophila to protect the transforming insect inside, softer and more transparent in the case of Aedes. In both cases the internal tissues, subjected to internal pressure, are still undergoing extensive reorganization. Even delicate handling can inadvertently damage the cuticle, leading to dehydration or damage to developing organs, which could be fatal for the insect or block correct development.

Encouraged by the positive results with Aedes, we tested the gel with another sample fundamental in developmental biology, *Drosophila melanogaster*. We wanted to test pupae that are living in different environments, mosquitoes in water and drosophila in a dry environment. We proceeded then by embedding 20 *Drosophila* pupae in a hydrogel mesh in a 6-wells plate, treatments and control groups were the same as with the mosquito pupae. After imaging (Fig. S10), the pupae in the multi-well plate were again freed by simple pipetting up and down with water and returned to separate vials in the insectarium to test whether they would develop to full-grown, normal adults. *Drosophila *pupae, maybe due to the harder and more opaque cuticle, had a higher survival rate, with 78% for the gel-embedded pupae and 70% for the pupae experiencing the maximum level of combined toxicity (please see video S3 and Fig. S11), with a 90% survival rate for pupae that never left the vivarium. This represents respectively a 12% and a 20% drop in the survival rate as compared to pupae that never left the vivarium. While this is a substantial drop, it represents a significant improvement over current embedding gels. In fact, in contrast to the most widely used embedding media in microscopy, agarose, it is possible here to recover the living sample without the mechanical damage that can be caused by forceps or thermal damage due to the high temperatures needed to re-melt the agarose gel^10^. Embedding of the samples can allow microscopic observation under different angles in a light sheet setup to record the expression of a fluorescent transgene during development. Furthermore, the use of techniques with a lower photon budget compared to confocal will most probably help to increase the global survival of the sample owing to the decreased light exposure.

### Hydrogel characterization and performance as embedding medium for light microscopy

 Apart from large specimens imaged at low magnification, we also realized that standard diffraction-limited confocal as well as super-resolution STED microscopy can benefit from novel sample preparation techniques employing the properties of our hydrogel.

In super-resolution microscopy, the use of either aqueous (e.g., for Single Molecule Localization Microscopy (SMLM)) or hardening embedding media like Mowiol ^®^ (Hoechst, Frankfurt, Germany) (e.g., in STED Microscopy) is common. Hardening embedding media can be used for example to embed adherent and suspension cells for imaging. However, the sample is compressed along the z-axis during the hardening process using Mowiol or other hardening mounting media and thereby hampers the measurement of correct distances especially along the z- axis^22^. Liquid mounting media can be used for adherent samples instead^22^, however, imaging suspension cells at higher resolution requires still the proper immobilization of the suspension cells on the coverslip to avoid sample movement during the imaging period, for example by poly-L-lysine coating and centrifugation of the suspended cells onto a coverslip (Cytospin). In general, hydrogels can serve as embedding media for non-adherent samples without changing the sample’s dimensions (see also Flood et al.^5^). In addition, our hydrogel should be able to immobilize fiducial marker for drift correction in high- and super-resolution microscopy.

**Fig. 2:**
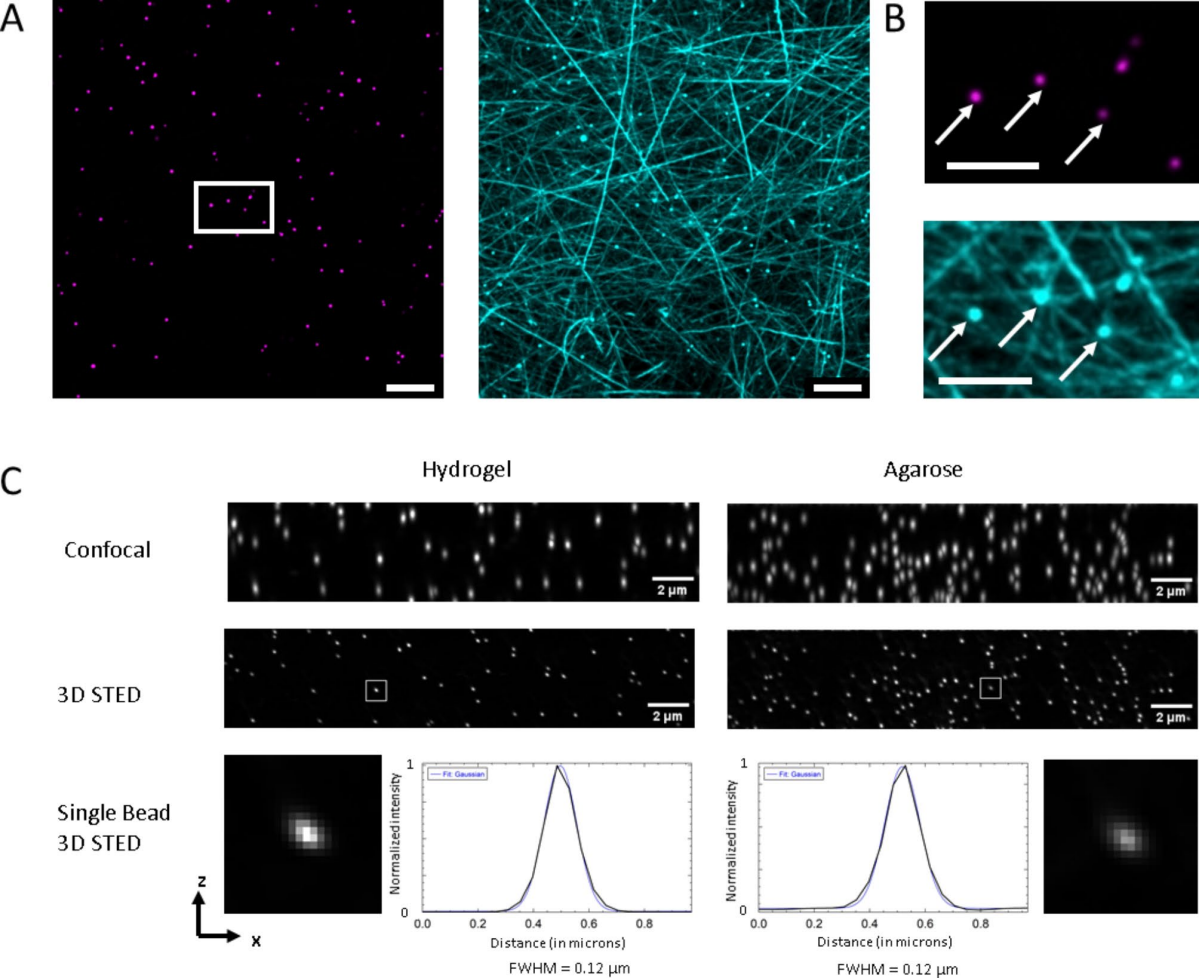
Fluorescent beads attach to hydrogel fibres and confirm the optical performance of the hydrogel for demanding 3D-STED super-resolution measurements. Confocal image of 100 nm multi-fluorescent Tetraspeck beads connect to Nile Red stained hydrogel fibres. (**A**) Maximum z-projection covering 6.2 μm height of hydrogel. Tetraspeck beads imaged with 488 nm laser excitation and green emission was detected (left, magenta). Hydrogel stained with Nile Red and the orange red fluorescence of Tetraspeck beads were recorded simultaneously while being excited with 561 nm laser light (right, cyan). Scale bar: 10 μm. (**B**) Enlarged view of the marked area in (**A**); arrows are indicating selected beads. Scale bar: 5 μm. (**C**) Imaging of 100 nm Tetraspeck beads with confocal and 3D-STED microscopy confirmed the high optical properties of the hydrogel for high- and super- resolution imaging (referenced to agarose). The Full Width at Half Maximum (FWHM) is derived from the Gaussian fit of the central, horizontal line profile of the single beads displayed in the lower row. The 3D-STED resolution enhancement in XZ is displayed side-by-side to beads embedded in 1.5% agarose gel. Images were deconvolved for denoising and maximum projections are displayed in x–z-view.

We first tested if fiducial markers—in this case 100 nm fluorescent Tetraspeck beads—can be attached to the fibres of our hydrogel. The fibrils of the hydrogel were stained with Nile Red enabling us to determine the anchoring point of the fluorescently labelled beads (Fig. 2A,B). We then compared the optical properties for high- and super-resolution imaging of our hydrogel in comparison to 1.5% agarose by imaging 100 nm fluorescent beads (Fig. 2C). Both, our hydrogel as well as agarose displayed similar resolution enhancement in 3D-STED super-resolution microscopy (FWHM for agarose 139.0 nm ± 10.0 nm and for hydrogel 133.7 nm ± 10.8 nm) (Fig. S14). The resolution enhancement was also independent of the imaging depth for agarose as well as hydrogel confirming that the refractive index of the two gels is close to water (Fig S14). Using standard confocal microscopy, we compared the fluorescence intensity of 100 nm beads in hydrogel and agarose. Both gels displayed properties that could be considered comparable in practical terms (Fig. S15). In conclusion, both gels, our hydrogel and agarose, should be suitable for demanding STED applications.

**Fig. 3:**
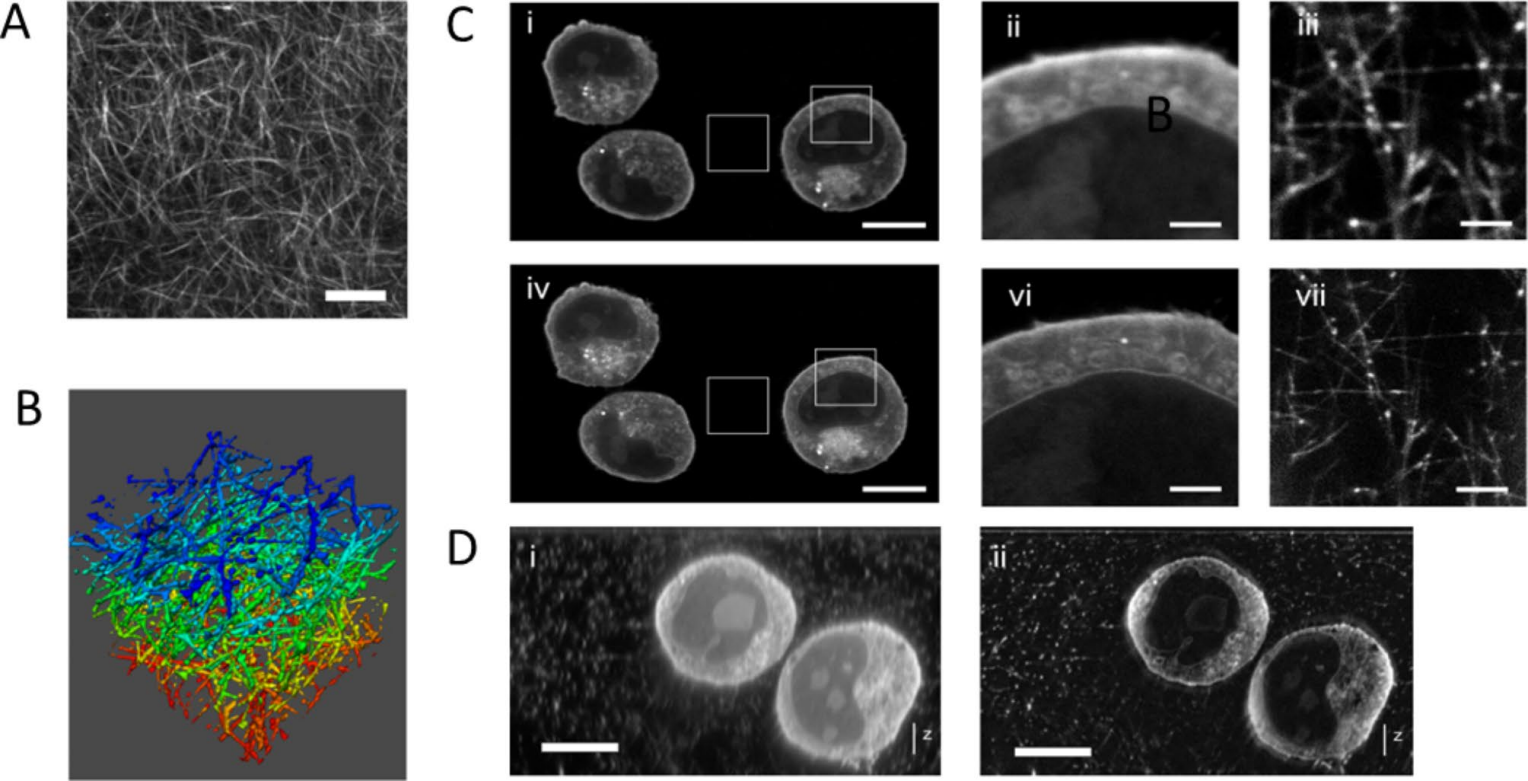
Characterization of the hydrogel by confocal and STED-microscopy and imaging of immobilized suspension cells. The lipid portion of the hydrogel was stained with Nile Red and acquired with diffraction-limited confocal microscopy giving an overview of the homogeneity of the mesh (**A**, scale bar 10 μm). The fine 3D fibrillar mesh structure becomes apparent by near isotropic 3D-STED microscopy of a 11 μm × 11 μm × 11 μm cubic region (**B**, colour-coded 3D maximum projection red (0 μm) to blue (11 μm), deconvolved). Suspension cells embedded in hydrogel (**C**): confocal (upper row) and 2D-STED (lower row) images of Nile Red-stained and chemically fixed K-562 suspension cells (scale bars: 10 μm in (i, iv) and 2 μm in (ii, iii, v, vi). Insets are individually intensity- and contrast-enhanced enhanced in Fiji using the Brightness and Contrast function (“B&C”). Although cells and hydrogel are stained by Nile Red, the hydrogel remains invisible under standard imaging conditions (C (i and ii / iv and v)) compared to intensity-adjusted C (iii / vi). Typical photons counts in (**C**) were in the range of 100–250 photons per pixel for Nile Red labelled hydrogel and 2000–4000 photons per pixel for Nile Red labelled cellular membranes in both confocal and 2D-STED-images. XZ-scan of hydrogel embedded K-562 cells acquired with confocal (**D**, i) and 3D STED (**D**, ii). Non-linear gamma enhancement was applied using Fiji (“Gamma”, value 0.4) and background removed (“Subtract Background” with a radius 50) to display cells and hydrogel in one image. (Scale bars: 10 μm).

First, we further tested the compatibility of the hydrogel with STED microscopy by again staining the lipid groups with the fluorogenic dye Nile Red and imaging the mesh structure first with diffraction-limited confocal microscopy (Fig. 3A) and then with near-isotropic 3D-STED microscopy to image a 3D sub-volume of the hydrogel (Fig. 3B).

Next, we turned to biological samples and used K-562 bone marrow cells grown in suspension for high resolution confocal and super-resolution STED- imaging. The chemically fixed cells were applied to the glass coverslip by simply mixing them with the non-polymerized monomers and then the hydrogel was polymerized as a thin layer on the coverslip. We again used the fluorogenic membrane dye Nile Red to label the plasma membrane as well as interior membranes (Fig. 3C–D). Nile Red was kept in solution to enable constant dye exchange and thereby large-volume STED imaging^23^. Nile Red weakly labels the lipids / fatty acids of the hydrogel (see also Fig. 2); the fibres could therefore be visualized highlighting the mesh size of the hydrogel. Please note that the intensity of the mesh is not interfering with the imaging of the biological structure (Fig. 3C–D). We consider the direct visualization of the hydrogel meshwork an important advantage over other embedding media as providing a reference for evaluating the STED performance independently of the preparation and structure of a biological sample.

Encouraged by the results with the hydrogel, we embedded K-562 bone marrow cells in agarose and hydrogel and performed a side-by-side comparison (Fig. S16). The visual performance of both gels was—as it could be expected from the previous results—very similar for standard STED applications.

Next, we wanted to take advantage of being able to attach fluorescent beads as fiduciary markers to the hydrogel, which should enable drift correction for large volume imaging. We tested this correction approach successfully by aligning three colour STED images recorded with multiple STED depletion lasers lines (Fig. S6) and demonstrated that this can be also beneficial for large volume confocal imaging (Fig. S7) by using stably bound beads as reference structures.

To determine the stability of this meshwork over time, we took again advantage of Nile Red and imaged the network directly in a time-lapse video for over 25 min (Video S1). The dense networks of our hydrogel were stable and displayed only local and minor rearrangements over the observation period. Hence, our two-component gel can be stored for weeks and is a ready-to-use formulation which can be mixed with the sample at room temperature “on the bench” and solidifies in less than 5 min, making it an ideal candidate for 2D- and 3D-STED microscopy and enables novel and more efficient sample preparation protocols.

In addition to the standard confocal, a high-throughput platform (Cell Discoverer 7) and the STED microscopy setup, we also tried our gel in a traditional light sheet setup. Mechanical stability is the main challenge, as here the hydrogel where the sample is embedded is completely submerged in the liquid of the sample chamber. We therefore extruded a cylinder of gel from glass capillaries with blue tag, size 4, (Brand GmBH, Wertheim, Germany). The cylinder was submerged in water for one hour and recovered afterwards, the same was true for a similar cylinder of agarose at the same % weight (Fig. S8). This is not the case with some recent commercial advanced hydrogel formulations, like CyGEL (Biostatus, Shepshed, UK) and Noviocell (https://mechanocontrol.eu/noviocell-bv/). Mechanical resistance of the hydrogel was tested also for a longer period of time (24 h) with 300 µl of our hydrogel, as a droplet on a glass bottom dish and was recovered as solid after that time (Fig. S13).

## Discussion

There is a high variety of hydrogels used for microscopy of living specimens that can be roughly divided into two categories: mounting media for short imaging projects and growth matrices for long time-lapse acquisition. Many current applications and recent developments in microscopy rely on specific sample mounting techniques to achieve optimal results. Especially for live motile organisms/cells, care must be taken to use the right gel, as the mechanisms triggering gelation can have distinct impacts on different samples. When the organism must be kept alive during microscopic observation, hydrogels are the most used and effective embedding media, as they are transparent, keep the sample hydrated and have sufficient mechanical stability to keep the sample immobile.

When the need arose in the imaging pipeline for such embedding media, already existing hydrogels like agarose^7^ or Matrigel, a widely used 3D culture matrix for cell growth^24^, were used although neither hydrogel was created specifically for microscopy. We present here a second-generation hydrogel mesh capable of quickly and gently stabilizing the sample during imaging but with improved optical properties and low toxicity, granting the option of freeing the embedded organism from the mesh after observation without any damage. The cross linking of the fibres that confers stability to the gel is completed in times and conditions compatible with standard handling and imaging procedures. We have shown here that our hydrogel has suitable biocompatibility parameters to allow survival and development of the sample over longer times. The gel has sufficient stiffness to embed the living sample, but not impair its development and allows some degree of sample movement. The mesh of the gel also allows gas-nutrient exchange with the environment surrounding the gel. Of equal importance, we can easily release the sample from the gel matrix. We tested the hydrogel with pupae from two different species and we were able to immobilize and release them without artefacts due to compression or hypoxia as could be the case with stiffer matrices or using cover slips^25^.

Irregularities in oxygen supply for a living organism can cause malformations or death^16^, but in our case the majority of the pupae developed to full-grown adults. Considering now microscopy of fixed samples, while toxicity of the mounting medium is certainly less relevant, there could however be other unforeseen factors playing an important role in our ability to detect the dye, especially in the case of advanced application such as superresolution^26^. For this reason, our hydrogel was tested also with STED microscopy.

As expected from the optical key parameters, our hydrogel formulation has proven its worth in diffraction-limited light microscopy and particularly in a nanoscopy application (STED super-resolution microscopy of chemically fixed mammalian cells). First, the immobilization and embedding of suspension cells in a matrix abolishes the movement of the cells avoiding any distortion of the cell shape (e.g., due to partial attachment to the glass surface). Second, the introduction of small multi-colour beads into the hydrogel enables fast and easy alignment of three-colour STED images which provides a valuable simplification for multi-colour super-resolution imaging.

The minimum gelator concentration values required for gelation are in the same range as those of agarose (~ 1% w/w). The pH of gelation, currently set in a generic physiological interval centered at 7.3, can be adapted to the optimal pH required by the living organism to be immobilized. Stiffness is also customizable, ranging from a completely reversible gel-sol transition via mechanical agitation to more stable meshes.

An interesting development already possible starting from the original formulation of the hydrogel^18^ could be the creation of a self-repairing gel for long time-lapses. By cultivating yeast into the gel together with the sample to be mounted, one can achieve a gradual and physiological local re-gelation of the matrix, with the creation of new fibrillar structures in time.

## Methods

Being a two-component gel, and to avoid pre-aggregation, fresh stock solutions were prepared for each experiment.

### Fly and mosquito strains

w1118 *Drosophila* stocks were used for these experiments and were raised at 25 °C following standard procedures. *Drosophila* pupae were exposed to experimental treatments and returned back to standard fly media to hatch into adults. The New Orleans strain of *Ae. aegypti* used were raised at 27 °C and 70% humidity under a 12 h dark and light cycle with dawn and dusk set at 30 min using standard procedures^27^. Pupae were returned to growth water, kept within 20 ml polypropylene tubes capped with cotton wool, and allowed to eclose.

### Material synthesis

The gelator was synthesised following the protocols of Angulo-Pachon and Miravet^18^. Full synthetic details and characterisation are described elsewhere (for clarity, the gelator is named CD-005 elsewhere)^19^. The chemical structure of the gelator is shown in Fig.1A. A precursor solution was prepared by adding to 50 mg of the hydrogelator to 20 mg of potassium carbonate and then dissolving in 5 mL of distilled water under stirring. To form a gel, 400 µL of the precursor solution was placed in a bijou tube and mixed with 1 mL of sterile buffer.

### Characterization

#### Light microscopy

 Beads embedded in 1% (w/w) hydrogel were visualised on a LSM 510 confocal microscope (Zeiss, Jena, Germany) with a 10X NA 0.45 objective. In the experiments with live samples, pupae and larvae from insects were mounted in the hydrogel on a multi-well plate and scanned using the confocal microscope. Time-lapse was set to test for the combined toxicity of the hydrogel and the photons with a scan every five minutes for one hour on a Cell Discoverer 7 (Zeiss, Jena, Germany), with a 5x NA 0.35 objective, Optovar = 2.0× in oblique transmitted light modality. Illumination power was 0.1% with an exposure of 5.4 ms and images were collected with an Axiocam 712 mono camera. Images were captured using Zeiss ZEN software.

#### STED-microscopy

 An inverted TCS SP8 3X microscope (Leica Microsystems, Mannheim Germany) equipped with a 86x/1.2 NA water immersion objective (Leica HC PL APO CS2 - STED White) was used. The microscope was controlled by LAS X (software version 3.1.5.16308). Fluorophores were excited with either 488, 561, 594, or 633 nm laser light derived from a 80 MHz pulsed White Light Laser (Leica Microsystems, Mannheim Germany) and the stimulated emission was performed with a 775 nm pulsed laser and a 592 nm CW laser (Leica Microsystems, Mannheim Germany). The fluorophore emission was collected with Hybrid Detectors (HyD, Leica Microsystems, Mannheim Germany) using a gate of 0.3-6 ns with respect to the excitation pulse for depletion with the 775 nm laser and 1 to 6 ns with the 592 nm laser. Images were recorded in photon counting mode. The microscope was equipped with an incubation chamber (constructed in-house at EMBL workshops) and constant cooling ensured a temperature of 22.5 ± 0.2 °C inside the incubation chamber. Detailed imaging parameters for each measurement are listed in Supplementary Table 1. Image deconvolution was performed with Huygens Professional (version 16.10.1p2, Scientific Volume Imaging, Hilversum, The Netherlands).

#### Cell lines, fixation and embedding

 Bone marrow derived K-562 lymphoblast cells (obtained from ATCC, # CCL-243) grown in suspension were pelleted by centrifugation in a cell culture centrifuge at 1000 g for 5 min. The cells were resuspended in 800µL DMEM and transferred to a 1.5mL test tube. Cells were pelleted in a table-top centrifuge at 1,000 g for 5 min. The supernatant was removed, and cells carefully resuspended in 100 µL 4% EM-grade PFA diluted from the 16% stock with PBS (#E15714, Scienceservices, Germany) and incubated at room temperature for 75 min. Cells were washed twice by centrifugation at 1,000 g for 5 min in a table-top centrifuge and resuspended in PBS. The cells were embedded in hydrogel by mixing the cell pellet with 100 µL gelator solution (containing 50 mg hydrogel powder and 20 mg of potassium carbonate per 5 mL water), transferred onto a 35 mm glass bottom dish (#P35G-0.170-14-C, MatTek, USA) and overlaid with a thin layer of TrisHCl buffer (1 M, pH 6.8, approx. 250 µL volume). The hydrogel polymerized within 5 min and the sample was washed twice with PBS. The sample was stained with 2 mL of 300 nM Nile Red in 150 mM Tris buffer pH 8.0 and the dye was kept in solution to allow permanent exchange^23^.

Fluorescence staining of adherent HeLa Kyoto cells with Wheat Germ Agglutinin (WGA) labelled with AlexaFluor594 (#W11262, ThermoFisher, USA), Concavalin A labelled with AlexaFluor488 (# C11252, ThermoFisher, USA) and SiR-tubulin (#SC002, Spirochrome, Switzerland) was performed in PBS according to the manufacturers recommended dilutions for 30 min. In brief, Concavalin A and Wheat germ agglutinin were used at a dilution of 1:1000 of the recommended stock solution and SiR-Tubulin at a final concentration of 1µM (1:1000 dilution of the 1mM DMSO stock). Cells were washed thrice with PBS for 5 min., overlaid with a layer of hydrogel gelator solution mixed with 100 nm Tetraspeck beads (1:50 ratio bead to gelator solution, #T7279, ThermoFisher, USA) and polymerized with TrisHCl buffer as described above.

#### Imaging of fluorescent beads in hydrogel

10 µL of 100 nm Tetraspeck beads (#T7279, ThermoFisher, USA) were mixed with 50 µL of gelator solution and added to the center of a 35 mm glass bottom dish (#P35G-0.170-14-C, MatTek, USA). 50 µL of 1 M MES solution (pH 5.8) were added to rapidly and homogenously polymerize the gel. After 5 min, 2.5 mL 100 mM TrisHCl buffer (pH 7.6) was added and after 5 min the sample was washed thrice with 2.5 mL TrisHCl buffer to return to a neutral pH and remove unbound beads before STED or confocal imaging was conducted.

#### Imaging of fluorescent beads in agarose

2% low gelling temperature agarose (#A9414, Sigma Aldrich, St. Louis, USA) prepared in ddH_2_O was liquefied in a heating block and kept at 50 °C. 50 µl of agarose solution were mixed with pre-warmed 16.7 µL of 100 nm Tetraspeck beads (#T7279, ThermoFisher, USA) and applied to the center of a 35 mm glass bottom dish (#P35G-0.170-14-C, MatTek, USA) and let it cool to room temperature for imaging.

#### Embedding cells in agarose

2% low gelling temperature agarose (#A9414, Sigma Aldrich, St. Louis, USA) prepared in ddH2O was first heated to 85 °C and then kept at 45 °C in a heating block. 50 µl of agarose solution were mixed with 50 µl of a cell suspension of formaldehyde fixed K-562 lymphoblast cells in PBS. The agarose cell suspension was immediately applied to the center of a 35 mm glass bottom dish (#P35G-0.170-14-C, MatTek, USA) and let it cool to room temperature. After 5 min, 2.5 mL 100 mM TrisHCl buffer (pH 7.6) containing 5µM NileRed was added and cells were stained overnight.

##### Rheology

 Rheological measurements were carried out using an Anton Paar Physica MCR301 rheometer. For the frequency and strain sweeps, a cup and vane (ST10-4 V-8.8/97.5-SN42404) system, with a measuring gap of 2 mm, was used so that measurements could be directly performed in the 7 mL Sterilin vials. Frequency sweeps were performed from 1 rad s^− 1^ to 100 rad s^− 1^ at a constant strain of 0.5%. Strain sweeps were performed from 0.1 to 1000% at a frequency of 10 rad s^− 1^.

For the time sweeps, measurements were again performed using a cup and vane (ST10-4 V-8.8/97.5-SN42404) system, with a measuring gap of 2 mm. 0.57 mL of the stock solution of the gelator was placed in the vial and the rheometer set up for the measurement. 1.43 mL of the TRIS HCl 1.5 M buffer was then carefully added by pipette into the vial and the measurement started. Time sweep measurements were performed at 25 °C. A constant frequency of 10 rad s^− 1^ and a strain of 0.5% was applied.

##### Transmittance measurements

 First, we embedded the same concentration of beads in 1% w/w low melt agarose, and in our hydrogel and compared the intensity of the beads. A visual examination confirmed that the beads embedded in our hydrogel were brighter than the beads in agarose, (See Supplementary Fig. S3 online). There was also no visible background autofluorescence in the gel, as in the nanoscopy case. To have a more quantitative measure of transmittance, we acquired fluorescence spectra comparing our hydrogel (400 µL gelling solution + 600 µL Tris HCl pH 6.8), with a cuvette filled with 1% agarose gel. Photoluminescence measurements were conducted on an Edinburgh Instruments FS5 under identical conditions (0.5 nm excitation slit, 1 nm emission slit, 1 cm cuvette, room temperature). The blank was distilled water. Measures were performed in triplicate. The absorbance of our hydrogel was always lower or equal to that of the low-melt agarose gel (See Supplementary Fig. S4 online). Finally, we measured the refractive index with a refractometer ABBE 5 (Thermo Fisher Scientific, Waltham, USA) in different phases of gelation, finding a constant value of *n* = 1.37.

## Electronic supplementary material

Below is the link to the electronic supplementary material.

Supplementary Material 1

Supplementary Material 2

Supplementary Material 3

Supplementary Material 4

Supplementary Material 5

## Data Availability

The datasets used and/or analysed during the current study are available from the corresponding author on reasonable request.
