# Supplementary material for: A biocompatible supramolecular hydrogel mesh for sample stabilization in light microscopy and nanoscopy
Source: Sci Rep. 2024 Nov 25;14:29232. doi: 10.1038/s41598-024-76661-x (PMC11589135; doi:10.1038/s41598-024-76661-x)
Supplement: Supplementary file 5 — Supplementary Material 5 [file 41598_2024_76661_MOESM5_ESM.docx]

**Supplementary Information-A biocompatible supramolecular hydrogel mesh for sample stabilization in light microscopy and nanoscopy**

Supplementary Figs. 1–8 and Supplementary Table 1.

**Figure S1.**  Time taken for gelation in seconds with the addition of 100, 200, 300 or 500 μL of Tris HCl 1.5M to 200μL of gelator.

**Figure S2.** (a) Rheological time sweep. (b) Strain sweep for the materials. The data presented are an average of 4 measurements with error bars representing the standard deviation. (c) Frequency sweep for the materials. The data presented are an average of 4 measurements with error bars representing the standard deviation. In all cases, the storage modulus (G′) is shown as black data and the loss modulus (G″) is shown as red data.

**
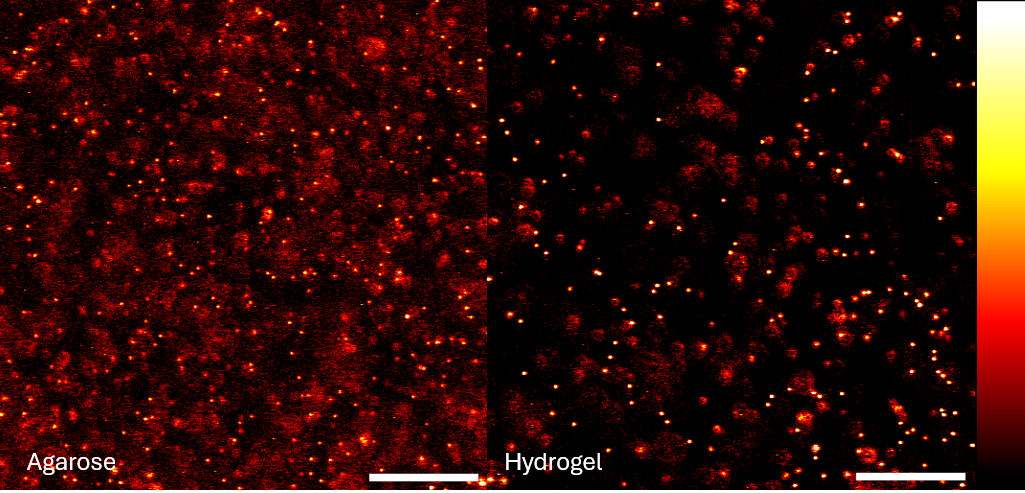
**

**Fig. S3. Comparative transmittance upon microscopy observation.** Beads in 1% (w/w) agarose gel visualised using a 10X objective on a Zeiss LSM 510 microscope, at 561nm and 1% laser intensity and same concentration of beads visualised in our hydrogel with same imaging conditions. Scale Bar 50 um, LUT range = 0 - 2500 AU

**
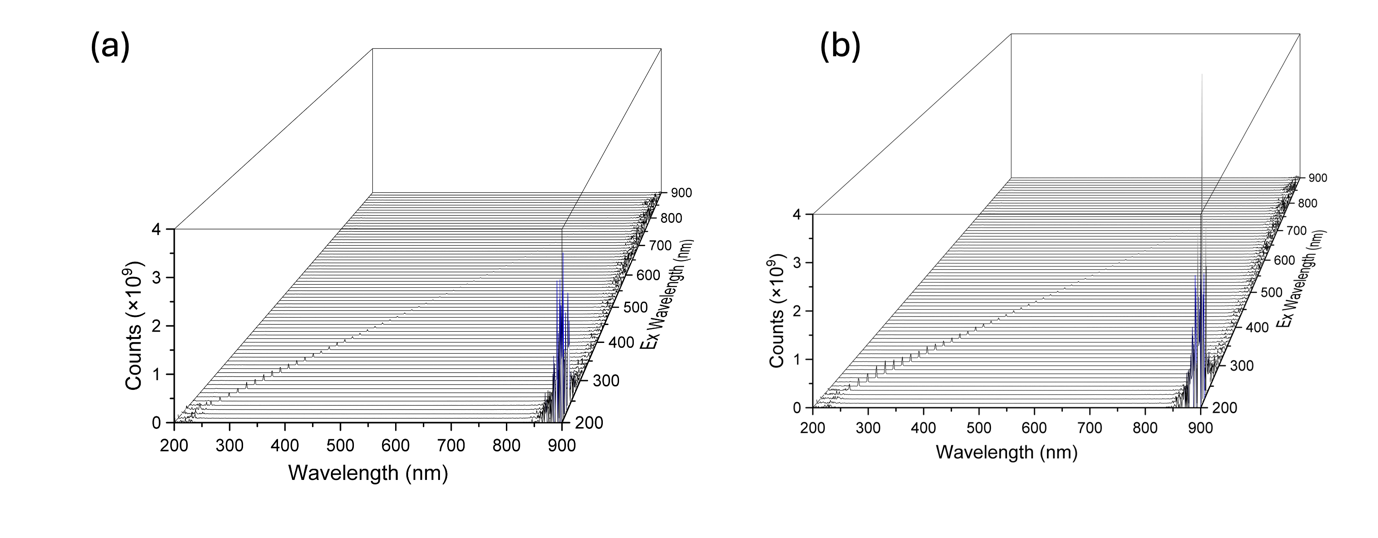
Fig. S4. | Quantitative fluorescence measured with spectrofluorimeter.**  (a) Emission map of an agarose gel (1% low melting agarose gel (6351.1) in DI water) showing no emission (only scattered excitation light). The excitation wavelength was varied from 200–900 nm with a 10 nm step; (b) Emission map of our hydrogel showing no emission (only scattered excitation light). The excitation wavelength was varied from 200–900 nm with a 10 nm step.


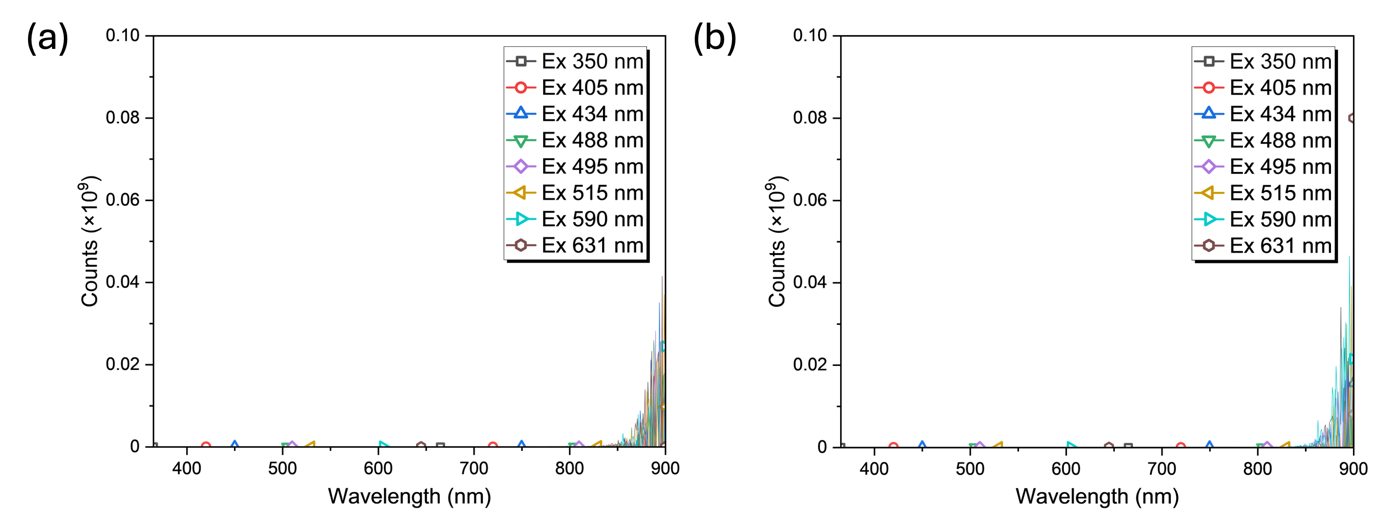


**Fig. S5. | Quantitative fluorescence measured with spectrofluorimeter at specific wavelengths.**  (a) Fluorescence emission of the agarose gel using standard biological imaging excitation wavelengths showing no emission at any of the excitation wavelengths chosen; (b) Emission of our gel using standard biological imaging excitation wavelengths showing no emission at any of the excitation wavelengths chosen.


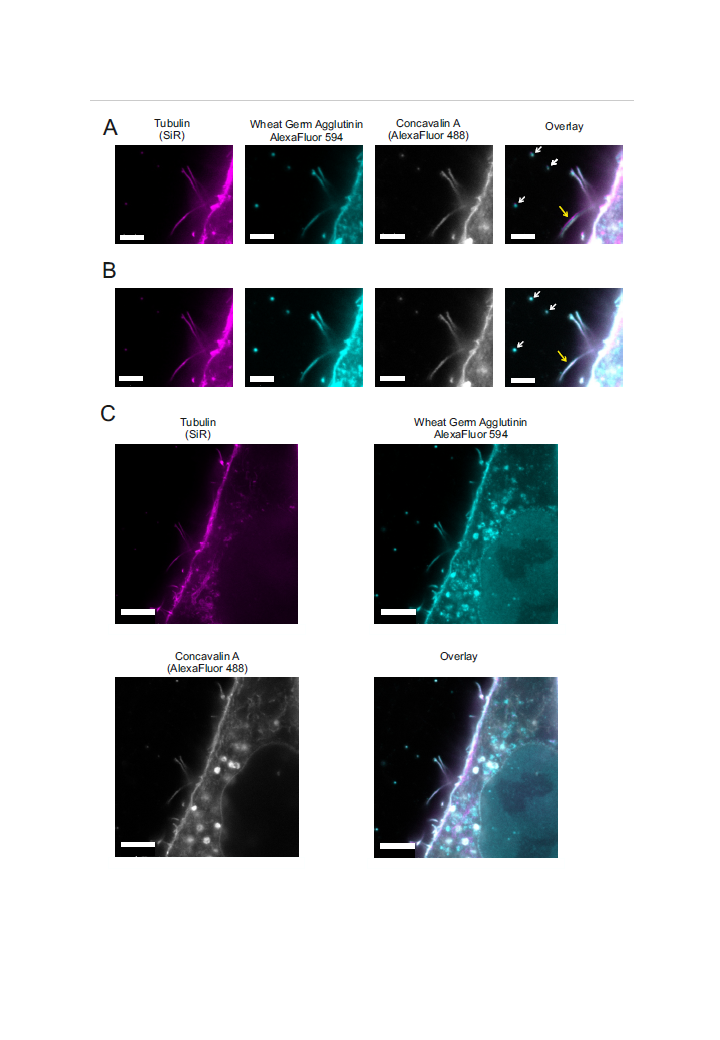


**Fig S6.** **Multi-colour STED drift correction by fiducials.** Adherent fixed Hela cells were overlaid with hydrogel containing 100nm multi-colour fluorescent beads. Individual 3D stacks were sequentially acquired with 2D-STED for three colours (SiR-Tubulin (magenta), Wheat- Germ Agglutinin (WGA) labelled with AlexaFluor 594 (cyan) and Concavalin A labelled with AlexaFluor 488 (white)) by using two depletion laser lines (775nm for SiR and AlexaFluor 594 and 592nm for AlexaFluor 488). The colour shift is clearly visible in a magnified region of a representative z-position (A, white arrows mark the position of 100nm multi-colour beads). The individual channels can be manually aligned (B, arrows) and the correction applied (C). Scale bars: 2µm (A, B) and 4 µm (C).


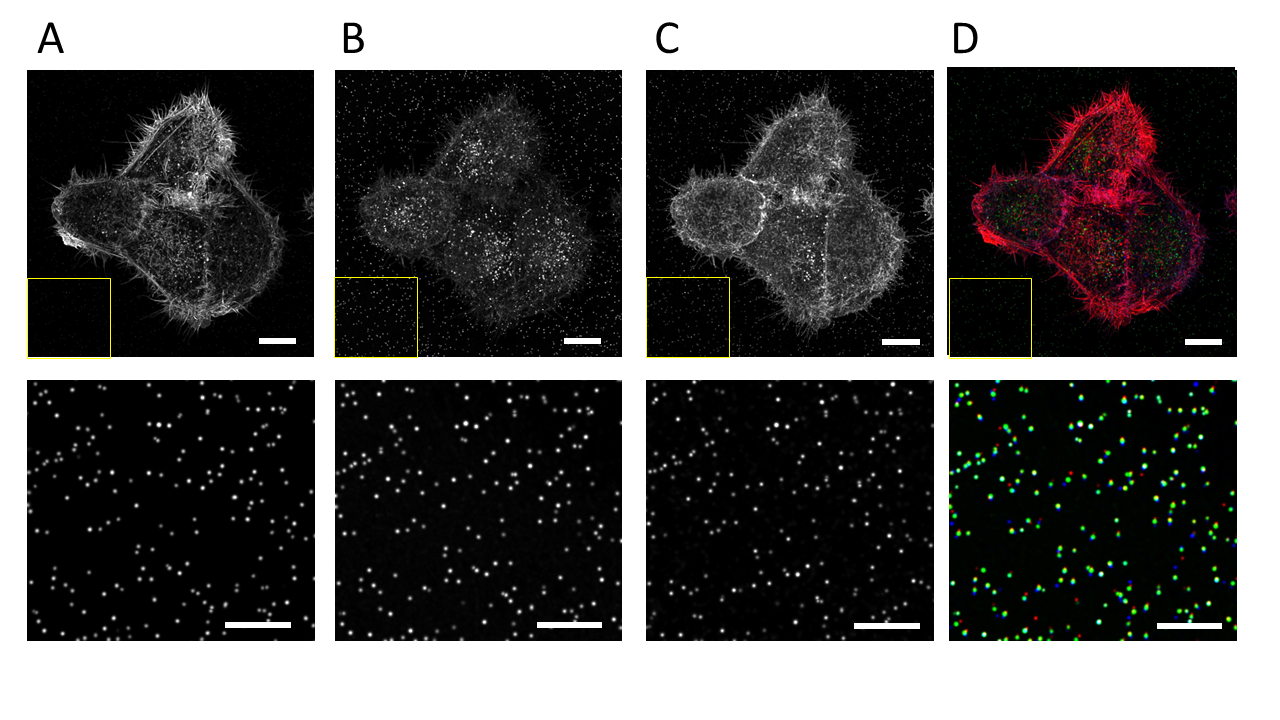


**Fig. S7.** **Hydrogel-bound fluorescent beads enable detection of thermal drift in large volume confocal imaging.** HeLa cells stained with (A) SiR-Actin (B) WGA-AlexaFluor594 and (C) Concavalin A-AlexaFluor488 were embedded in hydrogel containing four colour beads (100 nm Tetraspeck). Images are maximum z-projections of sequentially imaged stacks for each colour. Beads not colocalizing in the colour overlay (D) indicate drift in x,y or z during the imaging. Individual z-slices have been differently affected by drift indicated by different shifts between colours for individual beads. Beads only visible in one channel indicate drift in the axial direction at the edges of the stack. Overview of entire acquisition in the upper panel (Scale bar: 10 µm) and Zoom-in (as indicated in yellow) in the lower panel (Scale bar: 5 µm).


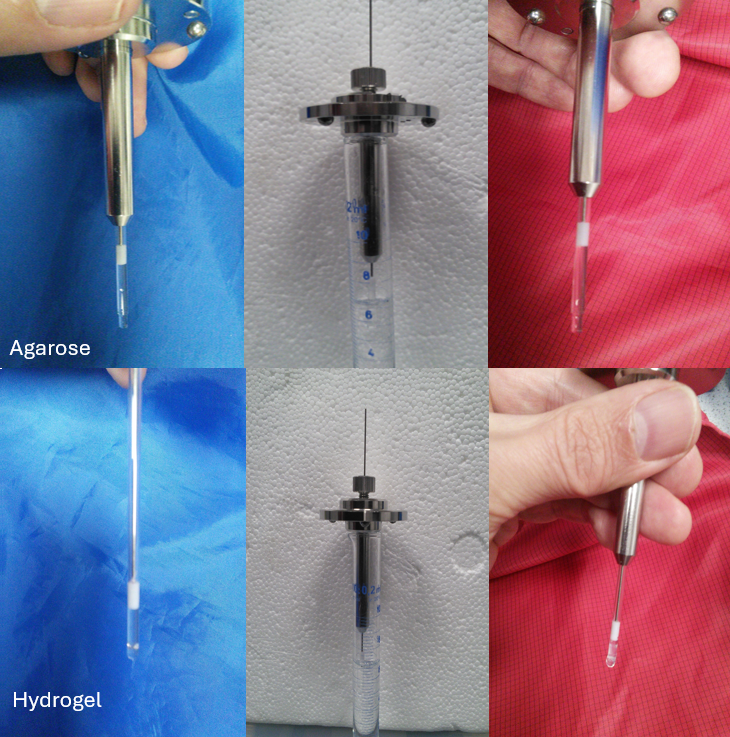


**Fig. S8.** Mechanical stability of agarose and hydrogel before, during and after 1 hour of submersion in distilled water.


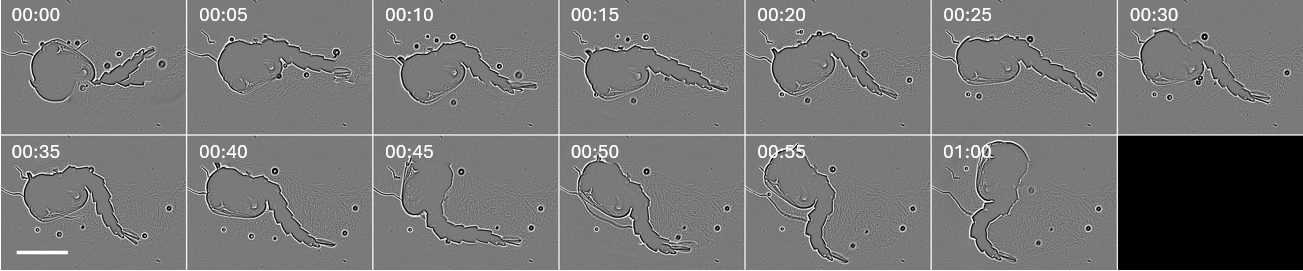


**Fig. S9.** Montage of one mosquito pupa imaged for one hour while embedded in the gel, with a picture every 5 minutes. Scalebar 200 µm.


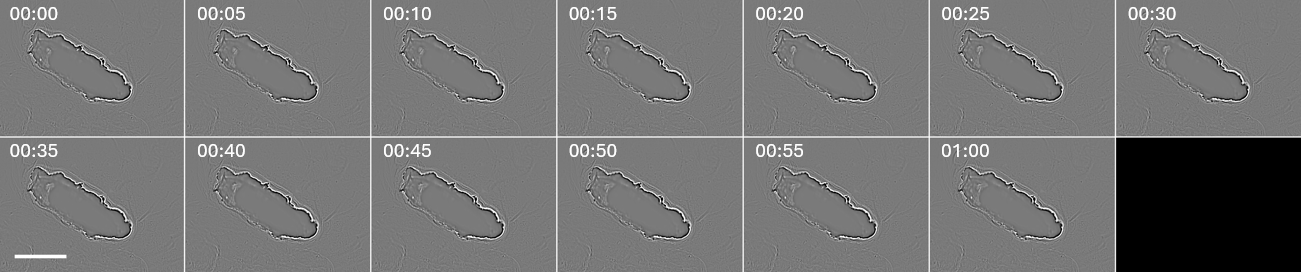


**Fig. S10.** Montage of one drosophila pupa imaged for one hour while embedded in the gel, with a picture every 5 minutes. Scalebar 200 µm.

**
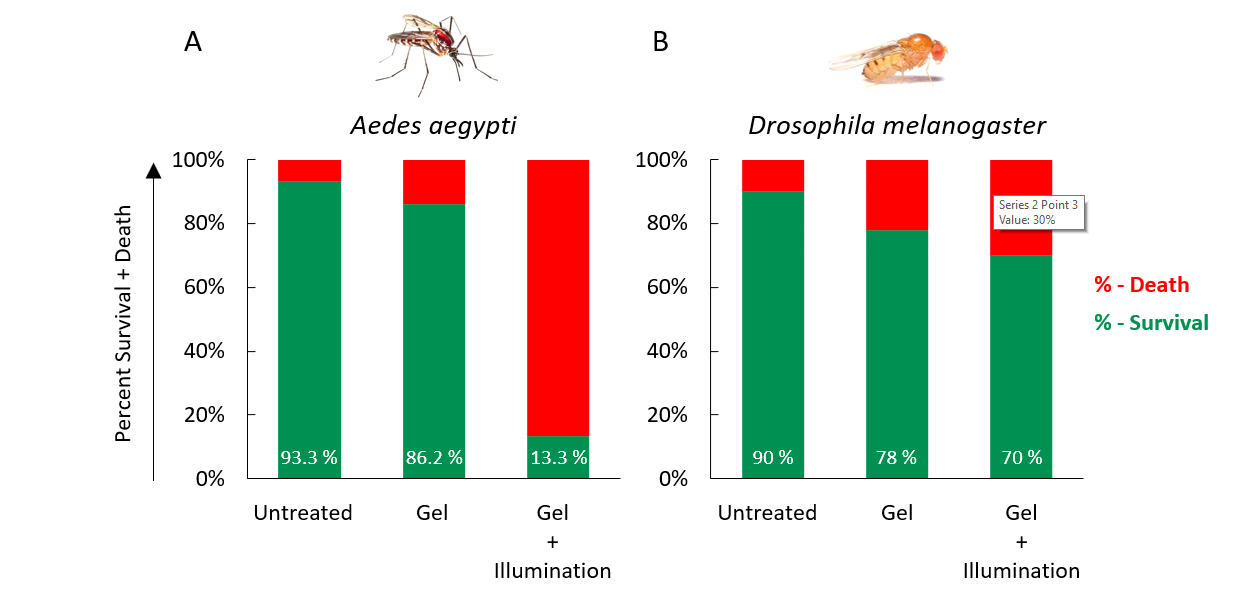
**

**Fig. S11.** Survival rate of pupae in different conditions.

Untreated - Insects that were never in contact with the gel and never left the vivarium.

Gel - Insects embedded in the vivarium, and freed after one hour in the Insectarium

Gel + Illumination - Insects embedded in the vivarium in LSTM/Drosophila facility, transported on campus to CCI, imaged with the microscope, returned to LSTM/ Drosophila facility, and freed after a total of over two hours embedding in gel to reach adult stage after eclosion from pupa.

**Fig. S12.**

Adult Aedes eclosing after being embedded as pupae in the hydrogel


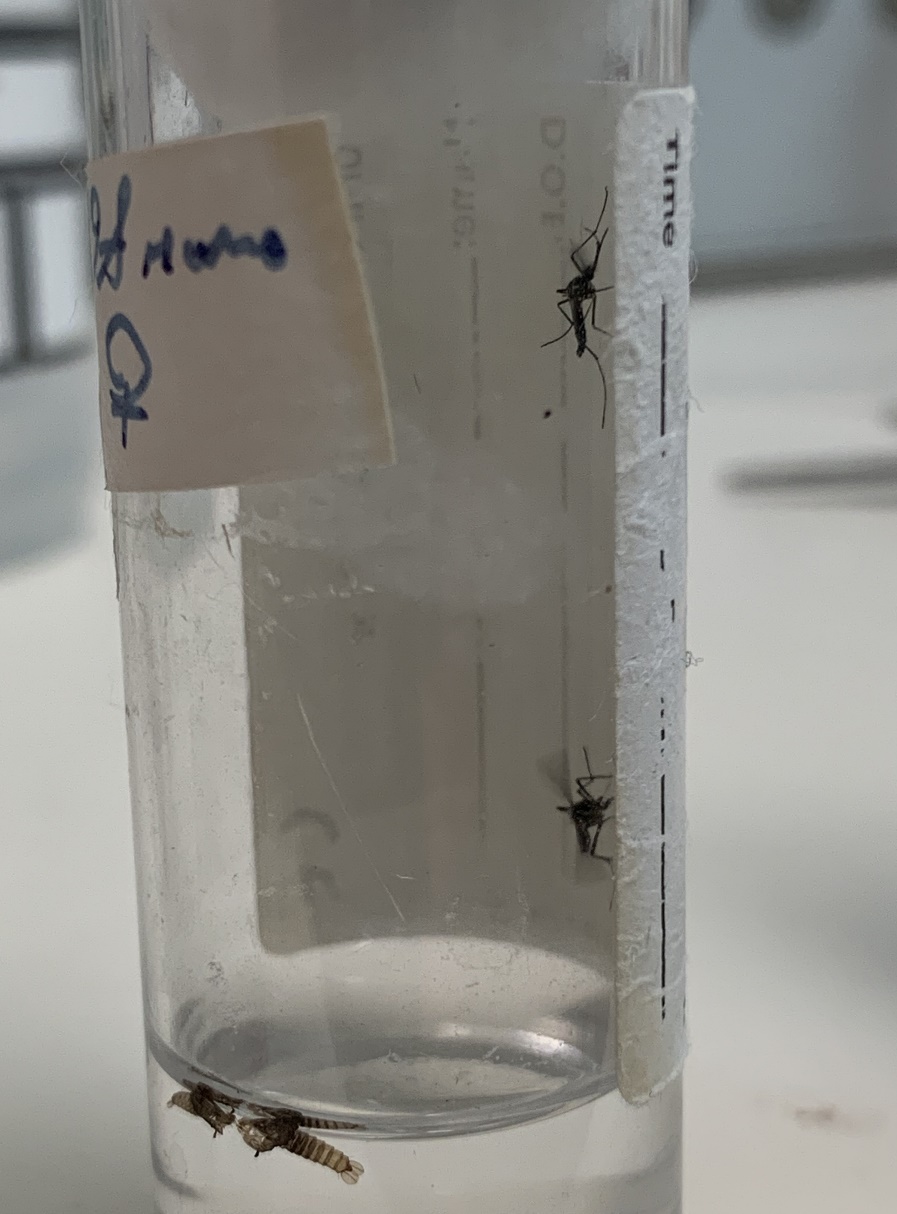


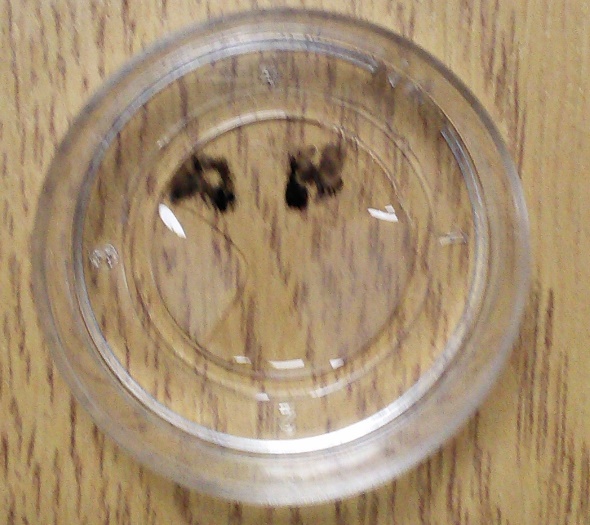


**Fig. S13**. Mechanical stability of a 300 µl droplet of agarose (labelled with “A”) and hydrogel ((labelled with “H”) with the same concentration deposited side by side on a glass bottom dish after 24 hours

_
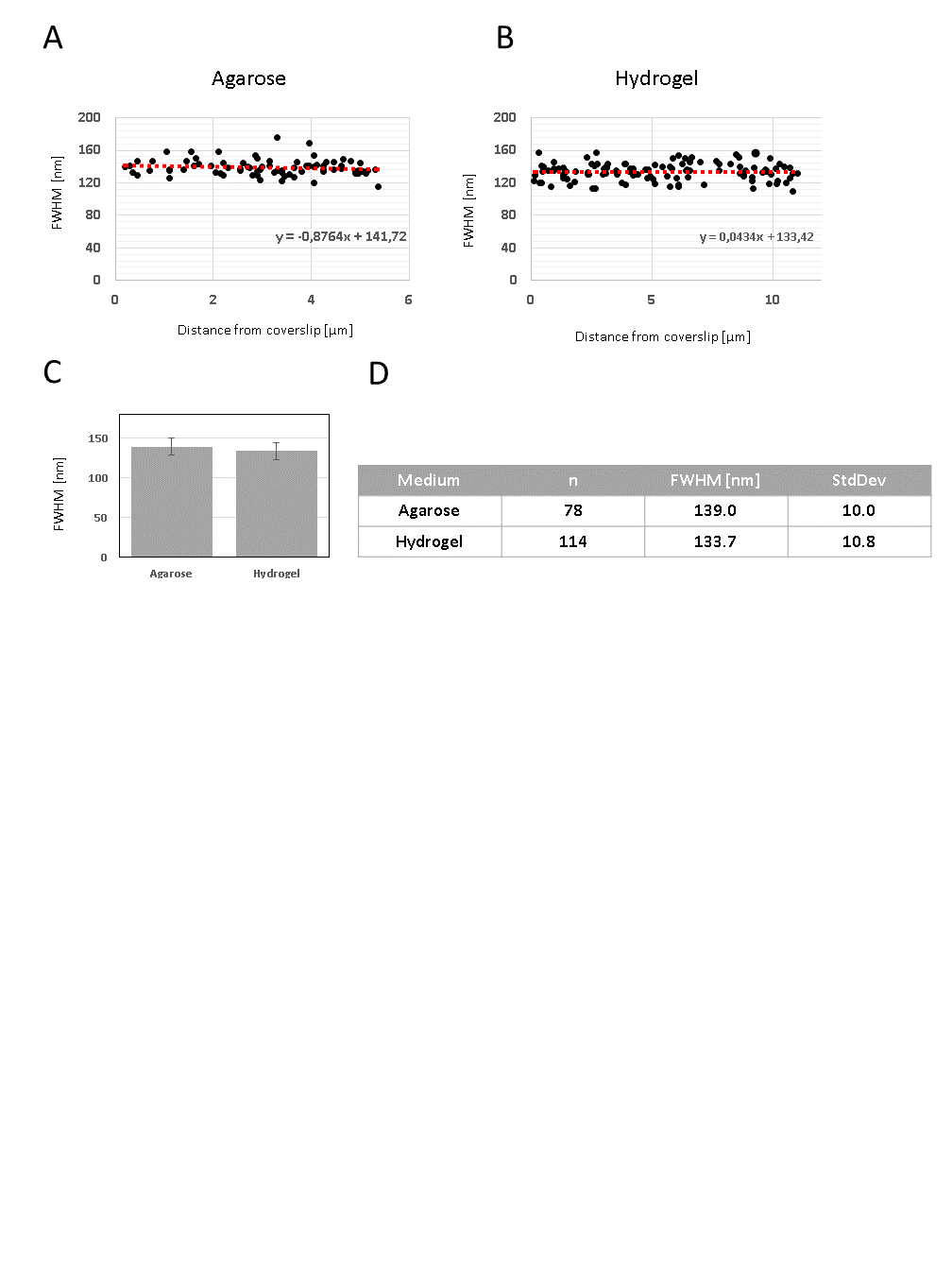
_

**Fig. S14.** 3D-STED of 100nm fluorescent beads in agarose and hydrogel: The FWHM was plotted as function of the distance from the coverslip as reference position. No depth dependent change could be detected (A). The average FWHM for agarose and hydrogel are similar (C, error bars are standard deviation) and the data is also summarized in (D).


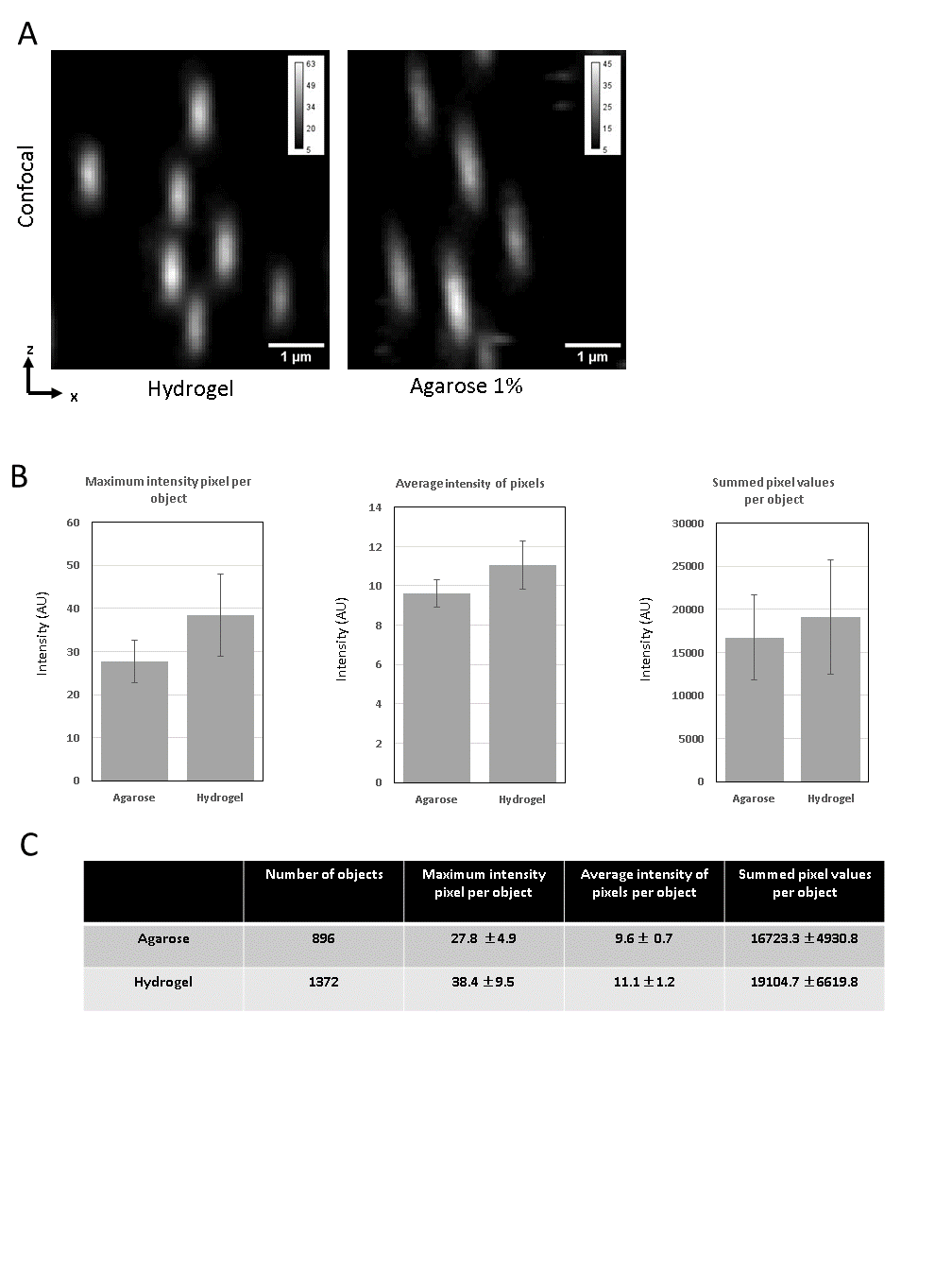


**Fig. S15.** 100nm beads were embedded in agarose or hydrogel and imaged from the coverslip up to 50µm depth using a confocal microscope with a 86x / NA1.2 water immersion objective. The position of the correction collar was optimized for each condition. Selected beads are shown as maximum projection in (A). Beads were automatically segmented using a watershed algorithm after applying a Gaussian filtering of the xz-stack. Intensity parameters where determined for each object. Results for the brightest pixel per object, the average intensity per object and the pixel sum per object are plotted with standard deviations (B) and summarized in table (C). Only objects with a volume in the range of a typical PSF were considered in the analysis and bead aggregates were excluded.

_
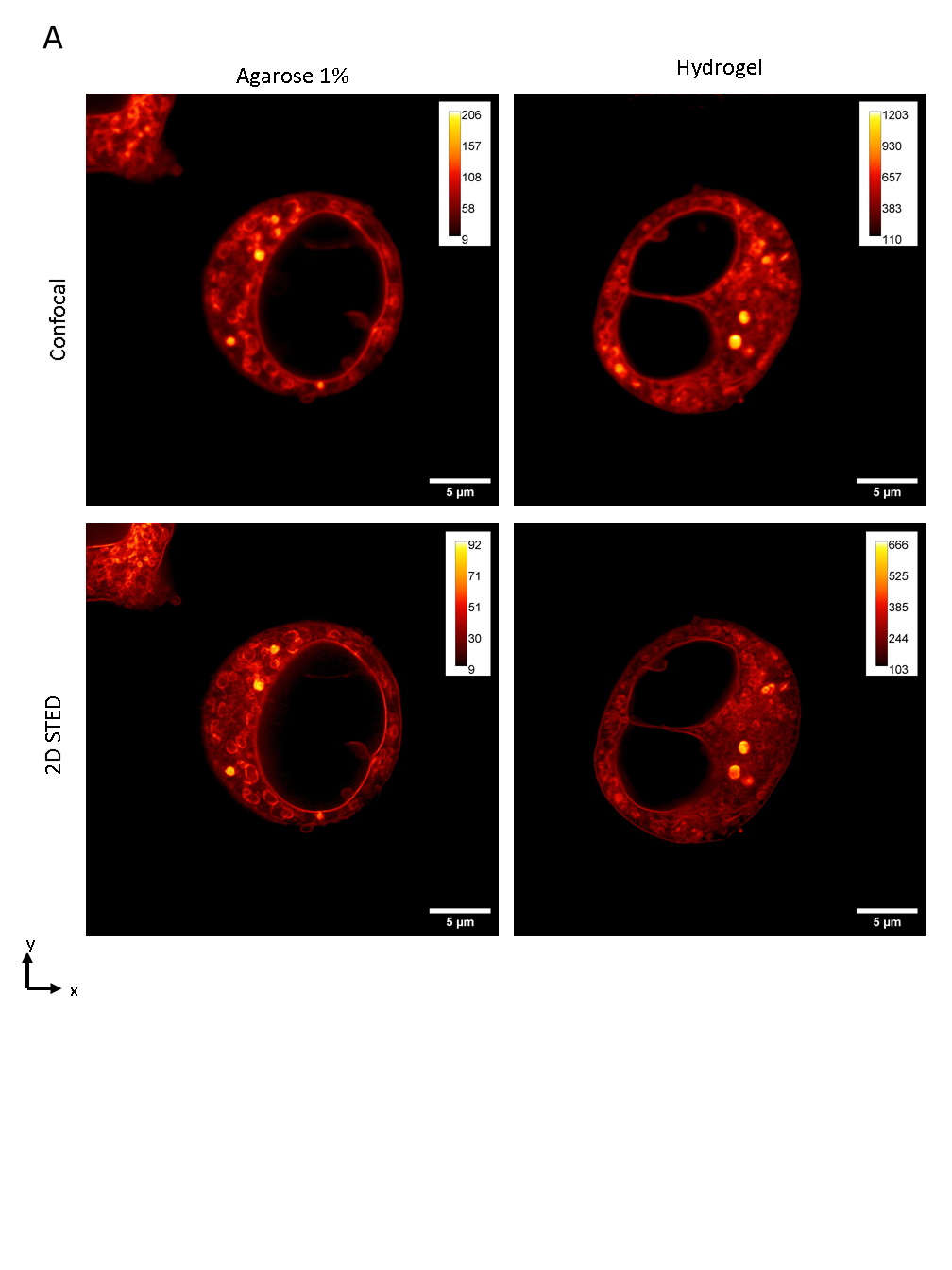
_

**Fig S16.** *(continued on next page)*

_
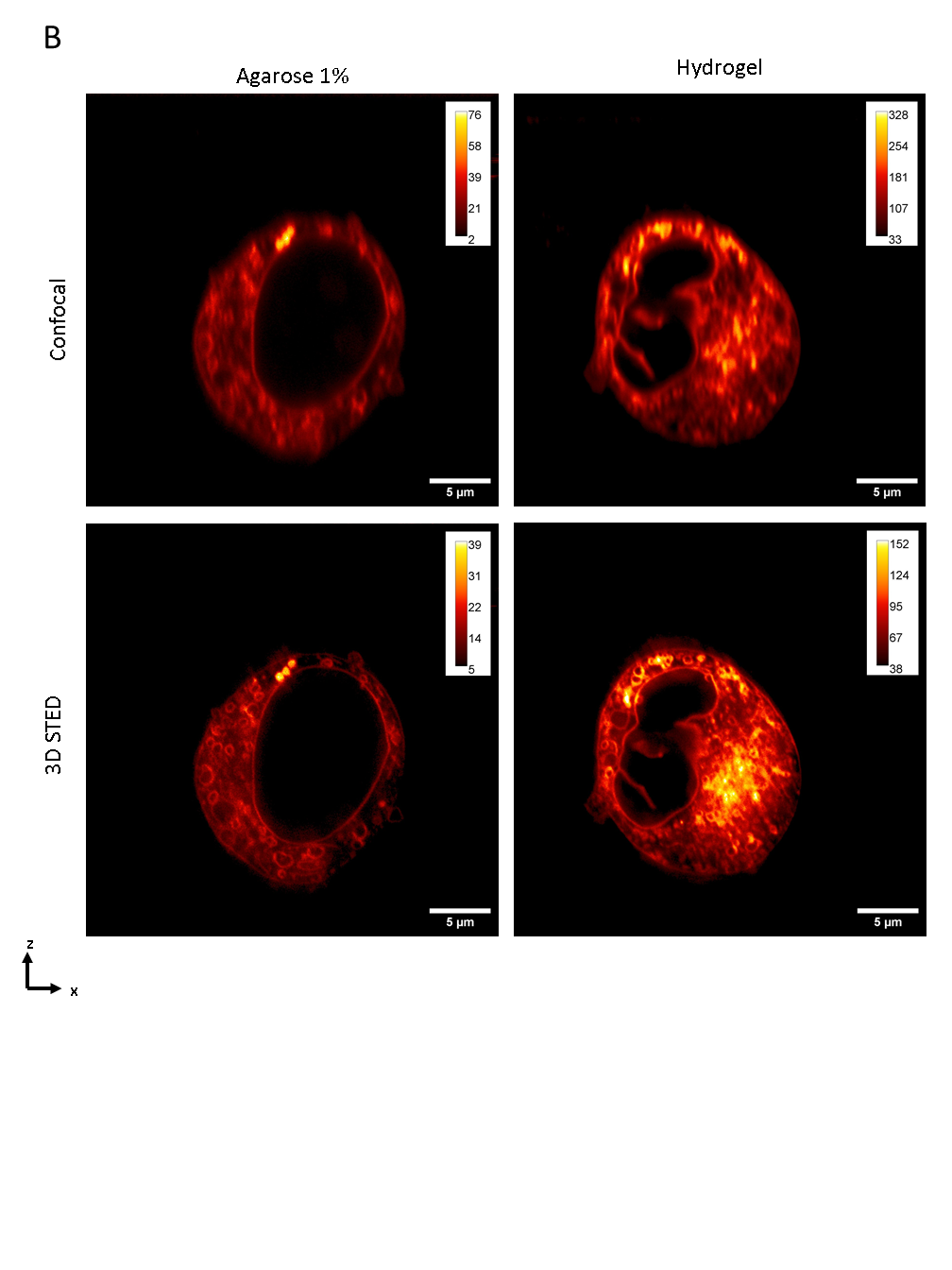
_

**Fig. S16.** NK532 cells embedded in 1% agarose (left) and hydrogel (right) were stained with 5µM Nile Red overnight (Agarose) or for 15 min (Hydrogel) and imaged under identical imaging conditions. Confocal images were recorded alongside the 2D-STED images (A) and the 3D-STED images (B).

**Video S1.** Time-lapse video of hydrogel fibres stained with Nile Red (1µM of dye in Tris-HCl buffer at pH 7.8). The gel was handled in the same way as the gels for Fig 2 except that no beads were added. The images series was filtered Gaussian filtered (“3D Gaussian Blur” with a two sigma for all axis in Fiji Version 2.14.0), drift stabilized in SVI Huygens (Version 23.10.0p6 with the default parameters) and corrected for bleaching (“Bleach Correction” in Fiji with “exponential fit”).Time is indicated in min:sec. Scale Bar: 5µm.
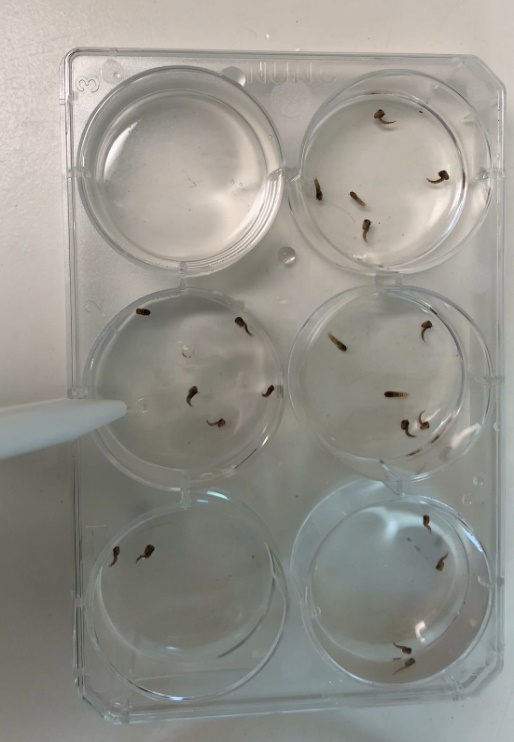


**Video S2.** Mosquito larvae freed from the hydrogel by simply pipetting water, resuming immediately normal motility.


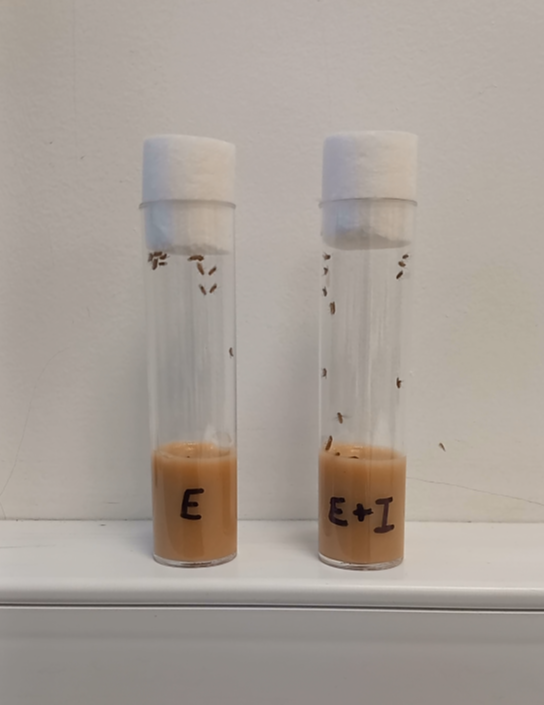


**Video S3.** Adult drosophila that eclosed from pupae that were only embedded in the gel in the vivarium (vial “E”), or embedded in the gel and imaged (vial “E +I”)

**Supplementary information on laser power for STED microscopy**

The laser intensities in Supplementary Table 1 are provided in % of total laser power as recorded in the meta data of the images. To allow comparison to other systems, typical laser output power are provided below. Measurements were performed after removal of the objective and placing a power meter on the microscope stage. Appropriate laser safety measure were implemented. The Leica WLL has a relative continuous spectrum allowing extrapolation of actual used laser intensities from the nearest measured line.

WLL intensities measured at 100% AOTF setting:

470nm: 0.37 mW

510nm: 0.32 mW

630nm: 1.0 mW

STED laser intensities (after laser alignment):

592nm: 550 mW

660nm: 480 mW

775nm: 450 mW

**Supplementary Table 1: STED- and Confocal Imaging parameters: see Excel file**
